# Supplementary material for: Pyruvate Dehydrogenase Kinase 1 inhibition mediated oxidative phosphorylation enhancement in cartilage promotes osteoarthritis progression
Source: BMC Musculoskelet Disord. 2023 Jul 20;24:597. doi: 10.1186/s12891-023-06585-6 (PMC10357736; doi:10.1186/s12891-023-06585-6)
Supplement: Supplementary file 1 — Additional file 1. [file 12891_2023_6585_MOESM1_ESM.zip › BP.Gsea.1653623667859/GOBP_CELLULAR_RESPONSE_TO_GONADOTROPIN_STIMULUS.html]

Details for gene set GOBP\_CELLULAR\_RESPONSE\_TO\_GONADOTROPIN\_STIMULUS[GSEA]

|  || Dataset | 55457\_collapsed\_to\_symbols.cls.cls #OA\_versus\_normal.cls.cls #OA\_versus\_normal\_repos |
| Phenotype | cls.cls#OA\_versus\_normal\_repos |
| Upregulated in class | normal |
| GeneSet | GOBP\_CELLULAR\_RESPONSE\_TO\_GONADOTROPIN\_STIMULUS |
| Enrichment Score (ES) | -0.56135076 |
| Normalized Enrichment Score (NES) | -1.7280728 |
| Nominal p-value | 0.009041592 |
| FDR q-value | 0.120020375 |
| FWER p-Value | 1.0 |
Table: GSEA Results Summary

  

Fig 1: Enrichment plot: GOBP\_CELLULAR\_RESPONSE\_TO\_GONADOTROPIN\_STIMULUS      
 Profile of the Running ES Score & Positions of GeneSet Members on the Rank Ordered List

  

| SYMBOL | TITLE | RANK IN GENE LIST | RANK METRIC SCORE | RUNNING ES | CORE ENRICHMENT || 1 | PAX8 | paired box 8 [Source:HGNC Symbol;Acc:HGNC:8622] | 3680 | 0.187 | -0.2463 | No |
| 2 | EDNRA | endothelin receptor type A [Source:HGNC Symbol;Acc:HGNC:3179] | 4663 | 0.099 | -0.3017 | No |
| 3 | SLC5A5 | solute carrier family 5 member 5 [Source:HGNC Symbol;Acc:HGNC:11040] | 5151 | 0.060 | -0.3270 | No |
| 4 | WT1 | WT1 transcription factor [Source:HGNC Symbol;Acc:HGNC:12796] | 5452 | 0.037 | -0.3425 | No |
| 5 | GATA6 | GATA binding protein 6 [Source:HGNC Symbol;Acc:HGNC:4174] | 5537 | 0.030 | -0.3428 | No |
| 6 | LHCGR | luteinizing hormone/choriogonadotropin receptor [Source:HGNC Symbol;Acc:HGNC:6585] | 5679 | 0.017 | -0.3502 | No |
| 7 | FSHR | follicle stimulating hormone receptor [Source:HGNC Symbol;Acc:HGNC:3969] | 5757 | 0.011 | -0.3538 | No |
| 8 | CYP1B1 | cytochrome P450 family 1 subfamily B member 1 [Source:HGNC Symbol;Acc:HGNC:2597] | 5925 | 0.000 | -0.3666 | No |
| 9 | EFNA5 | ephrin A5 [Source:HGNC Symbol;Acc:HGNC:3225] | 6997 | -0.080 | -0.4331 | No |
| 10 | CCNA2 | cyclin A2 [Source:HGNC Symbol;Acc:HGNC:1578] | 8047 | -0.159 | -0.4812 | No |
| 11 | GCLC | glutamate-cysteine ligase catalytic subunit [Source:HGNC Symbol;Acc:HGNC:4311] | 8534 | -0.196 | -0.4777 | No |
| 12 | PLAT | "plasminogen activator, tissue type [Source:HGNC Symbol;Acc:HGNC:9051]" | 8689 | -0.208 | -0.4458 | No |
| 13 | POR | cytochrome p450 oxidoreductase [Source:HGNC Symbol;Acc:HGNC:9208] | 10178 | -0.338 | -0.4902 | Yes |
| 14 | GCLM | glutamate-cysteine ligase modifier subunit [Source:HGNC Symbol;Acc:HGNC:4312] | 10439 | -0.366 | -0.4336 | Yes |
| 15 | GATA1 | GATA binding protein 1 [Source:HGNC Symbol;Acc:HGNC:4170] | 10958 | -0.425 | -0.3845 | Yes |
| 16 | INHBA | inhibin subunit beta A [Source:HGNC Symbol;Acc:HGNC:6066] | 11237 | -0.462 | -0.3091 | Yes |
| 17 | EDN1 | endothelin 1 [Source:HGNC Symbol;Acc:HGNC:3176] | 11276 | -0.468 | -0.2138 | Yes |
| 18 | NOTCH1 | notch receptor 1 [Source:HGNC Symbol;Acc:HGNC:7881] | 12555 | -0.776 | -0.1500 | Yes |
| 19 | NSMF | NMDA receptor synaptonuclear signaling and neuronal migration factor [Source:HGNC Symbol;Acc:HGNC:29843] | 12669 | -0.841 | 0.0179 | Yes |
Table: GSEA details [plain text format]

  

Fig 2: GOBP\_CELLULAR\_RESPONSE\_TO\_GONADOTROPIN\_STIMULUS      
 Blue-Pink O' Gram in the Space of the Analyzed GeneSet

  

Fig 3: GOBP\_CELLULAR\_RESPONSE\_TO\_GONADOTROPIN\_STIMULUS: Random ES distribution      
 Gene set null distribution of ES for **GOBP\_CELLULAR\_RESPONSE\_TO\_GONADOTROPIN\_STIMULUS**
